# Sustainable Materials for Engineering Applications

**DOI:** 10.3390/ma16186085

**Published:** 2023-09-06

**Authors:** Abdul Aabid, Muneer Baig

**Affiliations:** Department of Engineering Management, College of Engineering, Prince Sultan University, P.O. Box 66833, Riyadh 11586, Saudi Arabia; mbaig@psu.edu.sa

This newly added Special Issue (SI) of the *Materials* journal, titled “Sustainable Materials for Engineering Applications”, focuses on the foundations, characterizations, and applications of several sustainable materials. In recent years, there has been an increasing global focus on sustainability and the urgent need to transition towards environmental practices in various industries. Engineering, as a key sector driving innovation and development, plays a crucial role in shaping a sustainable future. This Special Issue aims to explore the latest advancements and challenges in the field of sustainable materials for engineering applications. Sustainable materials encompass a wide range of materials and technologies that minimize environmental impact while maintaining or even improving performance. This Special Issue welcomes original research, review articles, case studies, and perspectives that shed light on sustainable materials’ applications across different engineering disciplines. The objective is to provide a comprehensive platform for researchers, scientists, engineers, and practitioners to share their insights, exchange knowledge, and present cutting-edge solutions to address sustainability challenges.

The exploration and development of environmentally friendly materials involves a wide array of strategies, ranging from bio-based and recycled materials to those designed for improved end-of-life management. Extensive studies delve into the life cycles of these sustainable materials, yielding methodologies, tools, and practical case studies that rigorously assess their environmental impact and resource consumption. Innovative techniques are pivotal in sustainable material processing, with a focus on energy-efficient manufacturing, waste reduction, and pollution prevention, not only in improving the environmental aspects of material production but also by enhancing sustainability across industries. These sustainable material advancements extend to structural applications, exemplified by eco-friendly high-performance concrete, sustainable metals, composites, and polymers, striking a balance between impressive strength-to-weight ratios and environmental conscientiousness.

Renewable energy technologies greatly benefit from these materials, powering components in solar panels, wind turbines, energy storage, and fuel cells, and ultimately boosting efficiency and promoting cleaner energy sources. Sustainable materials are also transforming transportation through lightweight materials, electric components, and alternative fuels, significantly reducing the sector’s environmental impact. Additionally, in water treatment, tailored sustainable materials address water scarcity and pollution challenges, significantly enhancing the environmental performance of vital water management systems. The successful integration of these sustainable materials into engineering projects serves as a showcase of their potential, providing valuable insights into their benefits, challenges, and real-world applications. As industries increasingly prioritize sustainability, the continuous exploration and integration of such materials are vital for shaping an eco-conscious future. 

At present, there is an increased interest in replacing common engineering materials with sustainable materials due to the environmental challenges that our planet is exposed to. These challenges, encountered due to the use of common engineering materials, include (but are not limited to) changes in the climatic conditions, a decrease in available resources, and an increase in pollution. There has been a significant increase in the use of sustainable materials in the construction industry, as it poses a significant impact on the environment [1,2]. Studies have been performed to develop sustainable concrete materials to improve thermal insulation properties [3,4,5,6], and these materials are considered to be environmentally friendly and sustainable. Additionally, biopolymers are being considered as a replacement for polymers. These polymers are biodegradable and are considered to be sustainable materials in many engineering applications. Moreover, biopolymers obtained naturally have been gaining importance recently as they exist in abundance and are available from natural sources, which minimizes the impact on the environment and lowers costs [7,8]. 

It is well known that materials play a significantly critical role throughout their life span, in any product. However, their production poses several challenges. The traditional manufacturing processes are responsible for increasing carbon emissions, leading to a higher global warming concern. The presence of several studies in the literature related to the selection of materials highlights the importance of challenges involved with the proper selection of materials [9,10,11]. Currently, one of the most formidable challenges confronting humanity is the issue of global energy sustainability for the future. Concerns surrounding our environment, the depletion of finite natural resources, challenges in energy storage, environmental hazards, and the specter of natural disasters have collectively intensified our focus on the remarkable potential of sustainable energy materials. This pertains to their development, production, distribution, and packaging. As the demand for energy continues to surge, there is a pressing need to minimize adverse environmental effects while optimizing the utilization of thermal, electrical, optical, and chemical energy inherent in these sustainable energy materials [12]. Extensive research is being carried out in Lithium (Li) to develop Li-ion batteries as an energy storage device. In addition, in recent studies, Lithium-ion batteries have proven to be effective energy storage solutions for portable electronic devices and as energy sources in electric vehicles [13,14,15].

By bringing together diverse perspectives, this Special Issue seeks to foster interdisciplinary collaborations and promote the adoption of sustainable materials in engineering applications. It will serve as a valuable resource for researchers, scientists, professors, engineers, representatives, and participants committed to driving sustainable development and creating a more environmentally conscious future. Papers from the whole sustainable materials and manufacturing process, with high performance and energy efficiency, and referencing the experimental work, numerical simulation, mathematical expression, and optimizations techniques, with a focus on the anticipated and achieved results, are greatly welcomed.
